# The potential of a population register for addressing health inequities: an observational study using data linkage to improve breast cancer screening enrolment and participation in Indigenous Māori women in Aotearoa New Zealand

**DOI:** 10.1186/s12913-024-12186-3

**Published:** 2025-01-13

**Authors:** Phyu Sin Aye, Karen Bartholomew, Michael Walsh, Kathy Pritchard, Maree Pierce, Jenny Richards, Erin Chambers, Aroha Haggie, Jesse Solomon, Gabrielle Lord, Tiffany Soloai, Lorraine Symons, Roimata Tipene, Rawiri McKree Jansen

**Affiliations:** 1 Te Whatu Ora (Health New Zealand) Service Improvement and Innovation, Auckland, New Zealand; 2 University of Auckland, Auckland, New Zealand; 3 Te Whatu Ora Counties Manukau (Lead Provider), Auckland, New Zealand; 4 Te Whatu Ora Waitematā, Auckland, New Zealand; 5 Makaurau Consultants, Auckland, New Zealand; 6 ProCare Primary Health Organisation, Auckland, New Zealand; 7 National Hauora Coalition, Auckland, New Zealand; 8 Te Aka Whai Ora (Māori Health Authority), Auckland, New Zealand

**Keywords:** Breast cancer, Screening, Data linkage, Māori, Population register

## Abstract

**Background:**

Breast cancer screening in Aotearoa New Zealand (NZ) still has persistent inequitable coverage by ethnicity, especially for Indigenous Māori women. This project aimed to undertake systematic data linkage to identify and invite eligible Māori women to participate in breast screening.

**Methods:**

This is a cross-sectional observational study conducted in Northern New Zealand between 1/01/2020 and 30/06/2021. The BreastScreen Aotearoa (BSA) register was linked with the primary care data to identify and invite eligible Māori women (aged 45–69 years) to breast screening, who were not currently enrolled in BSA, and who were enrolled in BSA but had not been screened or overdue for breast screening. Invitations were sent through invitation letters, text messages and phone calls. Enrolment to BSA and screening participation at 15 and 18 months from the project’s start date were reported.

**Results:**

Through the data linkage, 2295 women who were not enrolled in BSA were identified eligible for breast screening. Approximately half (*n* = 1086) of the women were contactable within 5 contact attempts. Of these contactable, 345 (32%) women were enrolled in BSA, 421 (39%) were screened at 15 months, increasing to 441 (41%) at 18 months from the project start. Among women who were previously enrolled but never screened or overdue for screening, 1470 women were eligible. Their contact details were updated through the data linkage, which facilitated BSA to contact them successfully for breast screening. Consequently, 305 (21%) of these eligible were screened at 15 months, and increased to 332 (23%) at 18 months.

**Conclusions:**

The data linkage approach has evidenced the benefit of a population register to facilitate equitable access to breast screening services. Further work is needed on choices of combined approaches for optimising screening uptake equitably.

**Supplementary Information:**

The online version contains supplementary material available at 10.1186/s12913-024-12186-3.

## Background

Female breast cancer is the most commonly diagnosed cancer, responsible for 2.3 million new cancer registrations worldwide, and accounting for 11.7% of total cancers in 2020 [1]. Mortality from female breast cancer remains one of the leading causes of cancer death, contributing to 6.9% of total cancer deaths [1]. Screen-detection of breast cancer by mammogram in asymptomatic women yields substantially improved breast cancer survival. The breast cancer-specific survival rate of screen-detected group of women is 98% at 5 years and 95% at 10 years, while that of symptomatic group is 91% and 85%, respectively [2]. The national breast screening programmes are in place in many countries, with widely varying screening coverage; for example, > 70% in the northern European countries and < 20% in some countries in Latin America and Asia [3].

In Aotearoa New Zealand (NZ), BreastScreen Aotearoa (BSA) provides a national screening programme, which commenced in December 1998, offering biennial breast screening free of charge to 50–64 year old women, extended to include from 45–69 years in 2004 [4]. It covers multidisciplinary services including identification, invitation and screening mammography for eligible women, clinical assessment for screened women, referral to treatment, and counselling [4, 5]. BSA has eight Lead Providers (LPs) throughout the country that support individual primary care practices and women in their area with breast screening referral and recall. BSA currently does not have a population register for breast screening, and cannot systematically identify the eligible women, including those enrolled in primary care and those not enrolled. Therefore, breast screening participation relies on women being referred by their primary care provider or self-referring, if not being identified by LPs.

In NZ, breast screening has not achieved an equitable coverage by ethnicity, especially for Indigenous Māori women, with coverage consistently under the NZ target of 70% between 2016–2018 [6]. While Māori women with BSA screen detected cancers have equally positive outcomes to non-Māori [7, 8], overall there remain inequities in mortality for Māori women with a 33% higher chance of dying from breast cancer at 10 years after diagnosis, when compared to European women (the major ethnic group in NZ), based on the 2003–2017 report [2]. Late stage at diagnosis was the most important contributor to higher mortality in Māori women [9]. Breast cancer is also one of the ten commonest causes of avoidable death contributing to the life expectancy gap for Māori [10].

At the time of this project, the absence of a population register for breast screening has made it difficult to identify eligible women systematically and may have contributed to the current equity gap for Māori women. Despite a range of efforts over time, some LPs are still unable to work with all the general practices (or their organising bodies, Primary Health Organisations [PHOs]) in their area. The BSA National Policy and Quality Standards (NPQS) [11] allowed LPs to conduct local data matching with individual general practices if they specifically agreed to their patients being contacted by LPs. These often required a written agreement between the LP and the individual general practices, and there were many reasons noted by the LPs for why practices would not agree to data linkage; including that general practices were private businesses (government subsidised), that providers wanted to contact women themselves, and that they were concerned about privacy and confidentiality. LPs noted that it was resource intensive to create and establish relationships, and perform individual data matches, with individual practices. In addition, practice generated enrolment data may not be the same as centrally updated and verified enrolment data. In NZ, it is usual for general practice to invite women to their first screening (at aged 45) and to recall women for screening, although the BSA LPs also undertake invitation and recall.

Prior to the project, the BSA LPs had identified systematic, centrally conducted, data linkage as a key enabler to addressing equity, and agreed to undertake this project in the Northern region of NZ as a proof of concept and to support a case for investment in a population register. The area included four LPs delivering services delineated by geographic boundaries – at the time two district health providers (government delivered services) and one private provider (publicly funded). Our project aimed to undertake secure and systematic equity-focused data linkage to identify and invite eligible Māori women who have never been screened to participate in breast screening. The project was named the ‘500 Māori Women Campaign’ because approximately 500 Māori women were needed across the Northern region to be screened to reach the 70% coverage target at the time, creating a shared goal for the project.

## Methods

### Study design

This is a cross-sectional observational data linkage study carried out in the eligible population in the Northern region of New Zealand between 1 January 2020 and 30 June 2021. The reporting of this project followed the strengthening the reporting of observational studies in epidemiology (STROBE) statement (Supplementary Table 1) [12], and consolidated criteria for strengthening reporting of health research involving indigenous peoples (CONSIDER) statement (Supplementary Table 2) [13].

### Ethical considerations

#### Consent and approvals

The need for ethics approval was waived by the Health and Disability Ethics Committees; however, as part of the protocol preparation ethical issues were considered and documented, including mitigations, as part of good practice. These issues included privacy and confidentiality, the potential to cause distress (e.g., if contacting patients who had a recent cancer diagnosis or who had passed away), cultural safety and addressing inequities. Under the New Zealand Privacy Act (2020) and associated Health Information Privacy Code [14], the project was considered justifiable meeting an exemption (allowing waiver of consent for the project) because women who enrolled with primary care organisations sign a consent for offer of community services, including population screening. There was further support for this justification using the provisions for offer of service under Sect. 22F of the New Zealand Health Act [15], which allows health agencies to share data for the purpose of the offer of a health service. A formal Privacy Impact Assessment [16] was undertaken. The project was approved by the BSA Programme, the Ministry of Health Data Governance Group, the local Privacy and Security Governance Group (including legal and privacy representation), the Regional Privacy Advisory Group, the Metropolitan Auckland Data Stewardship Group, the Metropolitan Auckland Clinical Governance Forum, and district locality processes at each site. The participants were informed of the project at the time of contact, with the opportunity to decline enrolment or screening, and a standardised complaints process was agreed and documented should this be required. Our study was also guided by the Declaration of Helsinki ethical principles for medical research involving human participants [17].

#### Data management

The project used secure access to information held by BSA, general practices/PHOs, and the Ministry of Health to identify and contact the eligible participants. Electronic data files were transferred using the secure File Transfer Protocol (sFTP) the Ministry of Health approved electronic system and for the LP data sharing using the CitrixShare system [18] (a local district health approved secured file transfer mechanism); both mechanisms had been cloud risk assessed and penetration tested. Linked data was held in a separate partitioned part of the district health board IT system, only accessible to the one named project analyst.

#### Culturally safe approach (Māori oversight)

With the sensitive nature of both access to identifiable data (including contact details) and the breast screening service, data access and management were managed in a culturally safe and appropriate manner. Activities to ensure culturally safe management included the following: Māori membership of the project working group (seven of the 11 members of the group were Māori; this included membership from an urban Māori organisation Te Whānau o Waipareira that had a Memorandum of Understanding with the local health district, membership from a Māori PHO, Māori LP leadership, and Māori health commissioning leadership), Māori members of the relevant reviewing groups (who had a focus on ensuring cultural safety and equity benefit for wāhine Māori (Māori women) and on Māori data governance), review of the wording of the invitation letter including the use of Te Reo Māori (Māori language) by the district Māori health managers, a Māori equity assessment of the project via the localities process, and assessment of the project using the principals and project application tools of the Te Mana Raraunga Māori Data Sovereignty guidance [19]. Findings were reported back to relevant Māori organisations, the Māori GM Network and the Ministry of Health Māori Monitoring and Equity Group.

#### Communications

A project working group and regular reporting ensured good communication. While LPs have some differences in their usual processes of data linkage, referral and recall, for this project it was agreed to use common collateral including a standardised invitation letter, response scripting for privacy queries and a common complaints management process. All primary care providers in the Northern Region were notified of the project via Medinz, a communication platform, should they receive any questions from women about direct access by the LPs or data privacy.

### Study population

This project included women identified as Māori ethnicity, aged between 45 and 69 years, who were enrolled with a primary care practice in one of the following four Northern health district areas: Northland, Waitemata, Auckland, and Counties Manukau. This Northern region comprises diverse ethnic populations and approximately 40% of total NZ population [20]. Female Māori make up 15% (143,700) of female Northern Region population; within the 45 to 69 female age group, Māori make up 12% (33,300): 28% in Northland, 8% in Waitemata, 7% in Auckland, and 14% in Counties Manukau. The ethnicity data used were from the primary care enrolment record, routinely recorded in accordance with the Ethnicity Data Protocols for Health and Disability Sector of NZ [21].

### Data linkage process

The four LPs in the Northern region were involved in the project: North LPs (covering two districts), Central LP, and South LP; also supported by the central BSA team, in (at the time) the Ministry of Health (now in the National Public Health Service, Te Whatu Ora, Health New Zealand). The project analysts (Ministry of Health, health district and LP analysts) performed the data linkage centrally at the Ministry of Health between the BSA registers of those four LPs and the primary care enrolment age-sex-register of the Ministry of Health. Women’s enrolment to LP was based on their domicile area (address). Estimation of the primary care reach of the LPs using individual general practice local data linkage prior to project start was conducted by two project team members working with each LP to identify a list of all their individual practice data agreements and matching this with the PHO and named general practices within their geographic area.

Inclusion criteria: The data linkage systematically identified those eligible women 1) who were not currently enrolled in a BSA LP (i.e., not known to the LPs), 2) who were enrolled in a BSA LP but had not been screened or overdue (when eligible women had not been screened in the last 24 months) for breast screening (i.e., known to the LPs, but not able to be contacted, where this project could provide more up-to-date contact details to enable recontact). 

Exclusion criteria: Those women who were screened prior to the start of this project were excluded from this project invitation to breast screening.

### Invitation to breast screening

**Fig. 1:**
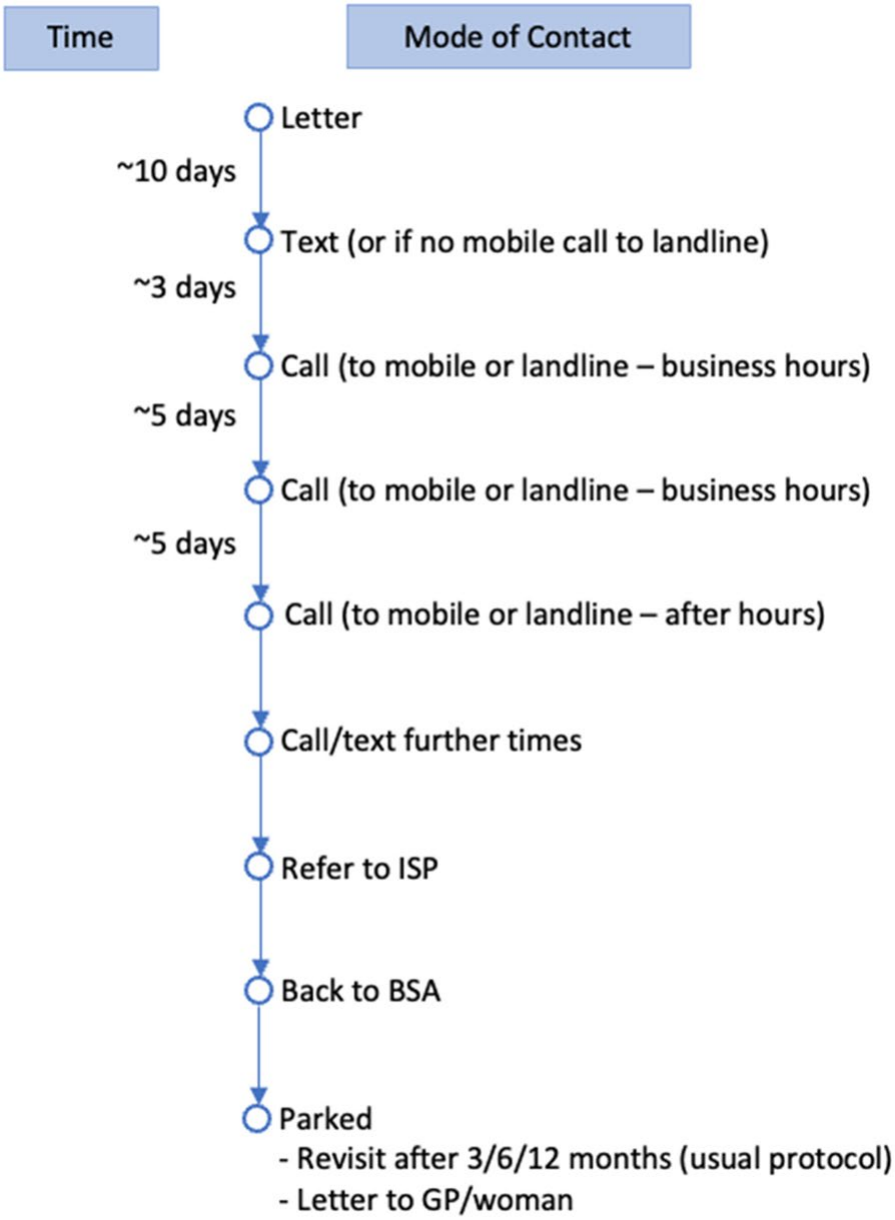
Contact timeframe. Note: ISP = Independent Service Providers. The timeframe in each step my vary; total length of the pathway was 5 days to 3 months

Once the women eligible for screening were identified, the respective local BSA LPs contacted and invited women to enrol in the breast screening programme and arrange a screening appointment through a letter and phone call process with standardised time points (Fig. 1). The LPs directly sent the invitation letters (Supplementary Table 3), followed by text messages and phone calls, to the eligible participants. Being able to contact within five attempts was defined as ‘contactable’, regardless of being enrolled or screened. The basis of five contact attempts, which is more than standard practice, is the equity focused nature of the project and success with this approach in other similar projects [22]. Women could enrol over the phone and book their screening appointment; wait time was days-weeks depending on when the appointment was convenient for the woman and the capacity of the LP. Of note, some of the LPs also have a standard practice, when they have details of potentially eligible women (particularly priority group women) [23], of keeping an active recall and reoffer approach periodically.

### Data analysis

The data of enrolment and screening participation between 1 January 2020 and 30 June 2021 was analysed, acknowledging the delays preceding the project initiation. Enrolment of women, along with the screening of participants, was considered to fall within this established project period. Outcome measures were systematically recorded at 15 and 18 months subsequent to the project's start date of 1 January 2020, when the data linkage had identified the eligible women, and the project commenced contacting and inviting the eligible women. Notably, a subset of participants was enrolled or screened before 1 January 2020 due to the project's delayed commencement. These cases were recorded as ‘prior to the project start’ and were excluded from the analysis of the project period outcomes.

The results were presented in numbers and percentages for each LP, separately for women who were not enrolled in a LP, and for those who were enrolled but never screened or overdue for breast screening. The marginal benefit of each contact attempt for screening was computed based only on successful contact attempts. The calculations were performed in MS Excel [24].

### Reporting of screening results

The outcomes of the project were reported back to primary care practices, with consent. An agreed template for reporting back to individual practices was developed with LPs and PHOs; reporting was based on aggregate data without individual level information. Higher level aggregate project reports were shared with all stakeholders.

## Results

The data linkage for this project involved four LPs: two (one in a large urban district and another one in a geographically dispersed rural/remote district) in the north, one central and one in the south of Northern NZ (Table 1). Prior to this project, there was clear variation by LP in how much pre-existing data linkage was occurring across their potential geographically bounded general practices: 18%, 44% and 83% of total general practices had agreements for regular data linkage with their North LPs, Central LP and South LP, respectively (Table 1).

**Table 1:** The status of general practice data linkage with their local Lead Providers (LPs) prior to project commencement

	**Number of practices with regular data linkage over total practices in that LP area**^**a**^	**Proportion**
North LP 1&2	18/102	18%
Central LP	59/134	44%
South LP	106/127	83%

^a^Denominator is an estimate of practices Lead Providers are expected to work with (some border general practices work with two LPs)

Through the data linkage, a total of 2728 women, who were not enrolled in a LP, were identified as eligible for breast screening (Table 2). After excluding 433 women who were screened prior to the project start, 2295 women remained eligible for breast screening, mainly contributed to by the younger aged women (< 50 years old group). Approximately half (*n* = 1086) of the women were contactable within 5 contact attempts, showing the highest proportion in Central LP (59%) and lowest in North LP2 (39%). Of those contactable, 345 (32%) women were enrolled in a LP at 15 months after the project start, 421 (39%) were screened at 15 months, and increased to 441 (41%) at 18 months. The proportions of enrolment and screening among different LPs were similar (Table 2).

**Table 2:** The results of breast screening data linkage for women who were not enrolled in a Lead Provider (LP)

	**North LP1**	**North LP2**	**Central LP**	**South LP**	**Total**
Eligible	843	605	493	787	2,728
Screened prior to start	103	83	106	141	433
Eligible (adjusted)					
Total	740	522	387	646	2295
Age					
45 to 49	449 (60.7%)	319 (61.1%)	216 (55.8%)	397 (61.5%)	1381 (60.2%)
50 to 54	120 (16.2%)	85 (16.3%)	72 (18.6%)	109 (16.9%)	386 (16.8%)
55 to 59	62 (8.4%)	49 (9.4%)	49 (12.7%)	75 (11.6%)	235 (10.2%)
60 to 64	71 (9.6%)	44 (8.4%)	34 (8.8%)	44 (6.8%)	193 (8.4%)
65 to 69	38 (5.1%)	25 (4.8%)	16 (4.1%)	21 (3.3%)	100 (4.4%)
**Contactable**^**a**^	**311 (42%)**	**203 (39%)**	**230 (59%)**	**342 (53%)**	**1086 (47%)**
Enrolled 15 months^b^	118 (38%)	63 (31%)	59 (26%)	105 (31%)	345 (32%)
Screened					
15 months^c^	131 (42%)	82 (40%)	88 (38%)	120 (35%)	421 (39%)
18 months^c^	137 (44%)	86 (42%)	92 (40%)	126 (37%)	441 (41%)

^a^Contactable determined by protocol – 5 contact attempts

^b^Percentages for enrolled and screened women are based on contactable women

^c^Some enrolled prior to programme start but were not yet screened hence larger screening numbers than enrolled

Of the total 2047 women who were previously enrolled, but never screened or overdue for screening, 577 women were screened prior to the project start, and were excluded (Table 3). Thus, 1470 women remained eligible for breast screening; half of them were < 55 years old. Following the normal contact process in place by LPs, after women’s contact details had been updated through the data linkage, 305 (21%) of those eligible were screened at 15 months (range among different LPs 13–26%), which increased to 332 (23%) at 18 months (range 14–28%) (Table 3).

**Table 3:** The results of breast screening data linkage for women who were enrolled in a Lead Provider (LP), but never screened or overdue for breast screening

	**North LP1**	**North LP2**	**Central LP**	**South LP**	**Total**
Eligible	927	227	222	671	2047
Screened prior to start	273 (29%)	84 (37%)	48 (22%)	172 (26%)	577 (28%)
Eligible (adjusted)					
**Total**	**654**	**143**	**174**	**499**	**1470**
Age					
45 to 49	122 (18.7%)	32 (22.4%)	32 (18.4%)	158 (31.7%)	344 (23.4%)
50 to 54	177 (27.1%)	44 (30.8%)	26 (14.9%)	146 (29.3%)	393 (26.7%)
55 to 59	171 (26.1%)	32 (22.4%)	25 (14.4%)	94 (18.8%)	322 (21.9%)
60 to 64	102 (15.6%)	20 (14.0%)	24 (13.8%)	67 (13.4%)	213 (14.5%)
65 to 69	82 (12.5%)	15 (10.5%)	67 (38.5%)	34 (6.8%)	198 (13.5%)
Screened					
15 months^a^	167 (26%)	19 (13%)	28 (16%)	91 (18%)	305 (21%)
18 months^a^	183 (28%)	20 (14%)	30 (17%)	99 (20%)	332 (23%)

^a^Percentage is of total eligible adjusted

**Fig. 2:**
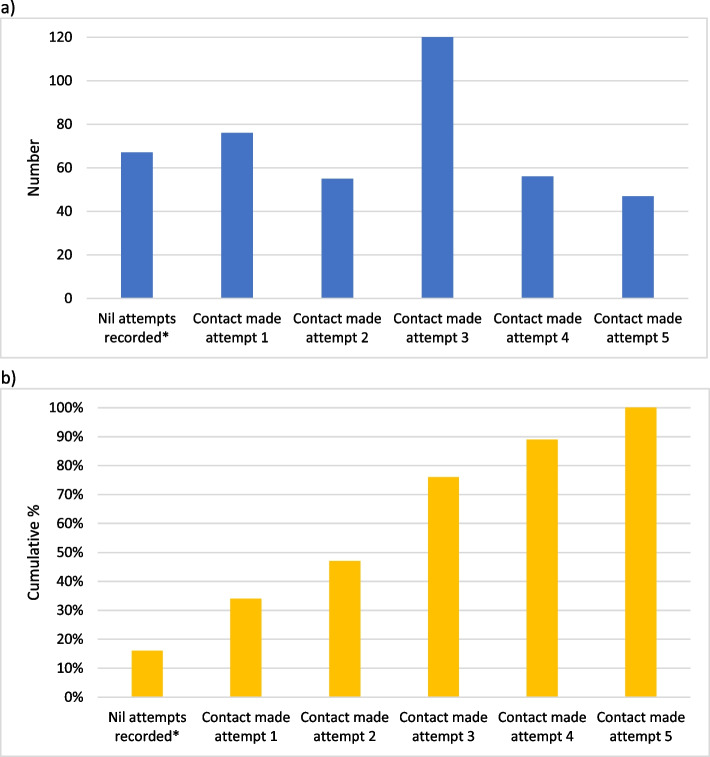
The marginal benefit of each contact attempt for women who were not enrolled in a Lead Provider, at 15 months, showing those screened (**a**) in number, (**b**) in cumulative percent. Note: These screened numbers were based on successful contact attempts. * These women were screened prior to any contact attempt of the project

In the group of women who were not enrolled in a LP, the marginal benefit of each contact attempt for screening analysis showed that 16% were screened prior to any contact attempt of the project; 18% were screened after successful contact at the first attempt; 13% after successful contact at the second attempt; 29% after successful contact at the third attempt; 13% after successful contact at the fourth attempt, and 11% after successful contact at the fifth attempt (Fig. 2). The timeframe for each contact attempt may vary, with the total length of the contact pathway of 5 days to 3 months (Fig. 1).

## Discussion

The data linkage in this project enabled Breast Screening LPs to successfully contact 1086 Māori women eligible for breast cancer screening, who were not previously known to the BSA programme. From this group of women now identified to the LPs, a total of 345 Māori women were enrolled, and 421 Māori women were screened at 15 months from the project start. (Of note, some enrolled prior to the project start but were not yet screened hence larger screening numbers than enrolled.) Additionally, 305 Māori women, who were previously enrolled in a LP but never screened or overdue for breast screening, were screened in this project where more up-to-date contact details were provided to the LPs for this group of women (where available). The result of 726 Māori women being screened overall exceeded the target of ‘500 Māori women’ project. Clear marginal benefit of additional contact attempts was demonstrated (up to 5 attempts recorded).

The project demonstrated baseline variation in LP ‘routine’ data linkage with primary care practices in their respective districts (from 18 to 83%). In NZ, general practices are publicly subsidised private businesses and can opt into or decline initiatives such as linking their enrolment register to the LP breast screening register to support the contacting of women who are not enrolled in breast screening. In some areas, practices have declined data linkage; LP representatives referred to reasons as including clinics where many patients choose private mammography (the data for which is not recorded in the BSA register), or they have concerns about privacy or data sharing, they have capacity issues or other priorities. This has led to a situation of variable data linkage occurring, and some women potentially missing out on the offer of free breast screening. The data linkage with the population-based register can fill this gap by systematically identifying the eligible women for breast screening and facilitate their participation in breast screening. The findings suggest that in the absence of a national population-based breast screening register, this systematic approach to data linkage could identify women not known to the programme, or with updated contact details, and this cohort of women had substantial uptake of the offer of screening (enrolment and participation). Participation was highest in the districts where ‘routine’ data linking approaches with primary care were the lowest, indicating the benefit of a systematic approach.

In our project, nearly half of the Māori women who were contactable were subsequently screened, demonstrating that many of those who were not currently enrolled in a LP were willing to participate in breast screening programme if they were offered the service systematically. This also demonstrated the potential benefit of a national population register to improve equitable access to breast screening services, providing a proof-of-concept for the Ministry of Health in the development of a population register investment case, with a government announcement that this would be funded in 2021 [25]. The benefit of the use of accurate and up-to-date population register has been recognised internationally for identifying individuals at risk and optimising participation in breast screening programmes [26]. The proportion of women who were contactable in this project was 47% among the eligible, which may be improved by regularly updating contact details in population registers. Further studies on the choice and combination of invitation approaches and timing may also be advantageous in optimising screening enrolment and uptake.

Screening for breast cancer is critically important to improve breast cancer outcomes equitably. The benefits of organised breast screening programmes depend on high levels of coverage of the population along with high quality screening and follow up services. Breast screening programmes achieving coverage of 70% eligible women (the NZ 2 year coverage target) can reduce mortality from breast cancer by approximately 30% for women who are screened, compared to those who are not [27]. Previous research reported that the mortality outcomes were not different between Māori and non-Māori women who had screen-detected breast cancer, whereas there remain marked inequities among those non-screen-detected cases (five-year breast cancer-specific survival rate 84.2% for Māori women versus 91.1% in New Zealand European women) [28]. Breast screening is one important intervention to reduce disease outcome inequities for Māori women [9, 28].

### Limitations

This is an observational project where other routine activity to promote breast screening and engage priority groups into the programme were ongoing through local LPs across the duration of the project. Enrolment and subsequent overall screening coverage may be impacted by the occurrence of COVID-19 [29–31], therefore the coverage over this period may not reflect the population level impact in the northern region. The data was linked for women who were enrolled in primary care, therefore those who were not enrolled in primary care may be missed. It is noted that the proportion of Māori who were enrolled in primary care was lower than that of non-Māori [32, 33], 87% Māori versus 96% non-Māori non-Pacific as at October 2020 [34]. However, non-primary care enrolled women would be identified in the new planned breast screening population register [23], similar to the National Cervical Screening Programme Register [35] and the Bowel Screening Register [36]. Challenges regarding complexities with primary care enrolment was also noted, where women can enrol outside of their domicile area, making data linkage difficult. It is also acknowledged that ethnicity data using any routine dataset; for example, the National Health Index (NHI) dataset and the primary care enrolment dataset, have known ethnicity data quality issues and levels of misclassification [37], which may also indicate a room for data quality improvement in the future.

## Conclusion

The data linkage approach has provided an enabler to improved participation in breast cancer screening programme. This project provides a proof-of-concept for the benefit of a national population register to enhance equitable access to breast screening services, and the findings contributed to the progress in establishment of a population register, which is now funded and underway. Māori women who had not been enrolled in the programme were willing to participate when they were invited to screening programme systematically. Further work is needed on choices of combined approaches including identification of eligible women, pathways and timing of contact, methods of invitation for optimising screening uptake equitably.

## Supplementary Information

Supplementary Material 1.

## Data Availability

The data used in the current project contain identifiable individual patient information. The data are not publicly available due to the data confidentiality and privacy restrictions but are available from the corresponding author on reasonable request and corresponding approvals. Please contact Health and Disability Ethics Committees at hdecs@health.govt.nz for ethics queries.
