# Publisher Correction: The potential of a population register for addressing health inequities: an observational study using data linkage to improve breast cancer screening enrolment and participation in Indigenous Māori women in Aotearoa New Zealand

**DOI:** 10.1186/s12913-025-12376-7

**Published:** 2025-02-10

**Authors:** Phyu Sin Aye, Karen Bartholomew, Michael Walsh, Kathy Pritchard, Maree Pierce, Jenny Richards, Erin Chambers, Aroha Haggie, Jesse Solomon, Gabrielle Lord, Tiffany Soloai, Lorraine Symons, Roimata Tipene, Rawiri McKree Jansen

**Affiliations:** 1 Te Whatu Ora (Health New Zealand) Service Improvement and Innovation, Auckland, New Zealand; 2 University of Auckland, Auckland, New Zealand; 3 Te Whatu Ora Counties Manukau (Lead Provider), Auckland, New Zealand; 4 Te Whatu Ora Waitematā, Auckland, New Zealand; 5 Makaurau Consultants, Auckland, New Zealand; 6 ProCare Primary Health Organisation, Auckland, New Zealand; 7 National Hauora Coalition, Auckland, New Zealand; 8 Te Aka Whai Ora (Māori Health Authority), Auckland, New Zealand

**Correction: BMC Health Serv Res 25, 64 (2025)**

**https://doi.org/10.1186/s12913-024-12186-3**

Following publication of the article, several corrections to the affiliations weren’t implemented.

- The details for affiliation 1 were incorrectly given as ‘Te Whatu Ora (Health New Zealand) Service Improvement and Innovation, University of Auckland, Auckland, New Zealand’ but should have been ‘Te Whatu Ora (Health New Zealand) Service Improvement and Innovation, Auckland, New Zealand’.
- ‘University of Auckland, Auckland, New Zealand’ should be a separate affiliation and added as affiliation 2 and should have been assigned to Phyu Sin Aye.
- Original affiliation 2 (Q4 Building Smales Farm, 78 Taharoto Road, Takapuna, Auckland 0622, New Zealand) should have been removed and all subsequent affiliations should have been re-numbered.
- The details for original affiliation 3 (new affiliation 1) were incorrectly given as ‘Te Whatu Ora Service Improvement and Innovation, Auckland, New Zealand’ but should have been ‘Te Whatu Ora (Health New Zealand) Service Improvement and Innovation, Auckland, New Zealand’.

The original article has been corrected and the publisher apologises to the authors and readers for the inconvenience caused by this error.

