# Supplementary material for: The potential of a population register for addressing health inequities: an observational study using data linkage to improve breast cancer screening enrolment and participation in Indigenous Māori women in Aotearoa New Zealand
Source: BMC Health Serv Res. 2025 Jan 13;25:64. doi: 10.1186/s12913-024-12186-3 (PMC11727381; doi:10.1186/s12913-024-12186-3)
Supplement: Supplementary file 1 — Supplementary Material 1. [file 12913_2024_12186_MOESM1_ESM.docx]

**Supplementary table 1. Strengthening the reporting of observational studies in epidemiology – the STROBE Statement**

|  | Item No | Recommendation | Page No |
| --- | --- | --- | --- |
| **Title and abstract** | 1 | (*a*) Indicate the study’s design with a commonly used term in the title or the abstract | 1 |
|  |  | (*b*) Provide in the abstract an informative and balanced summary of what was done and what was found | 2 |
| Introduction | | | |
| Background/rationale | 2 | Explain the scientific background and rationale for the investigation being reported | 2,3 |
| Objectives | 3 | State specific objectives, including any prespecified hypotheses | 3 |
| Methods | | | |
| Study design | 4 | Present key elements of study design early in the paper | 3 |
| Setting | 5 | Describe the setting, locations, and relevant dates, including periods of recruitment, exposure, follow-up, and data collection | 3,4 |
| Participants | 6 | (*a*) Give the eligibility criteria, and the sources and methods of selection of participants | 4 |
| Variables | 7 | Clearly define all outcomes, exposures, predictors, potential confounders, and effect modifiers. Give diagnostic criteria, if applicable | 6 |
| Data sources/ measurement | 8* | For each variable of interest, give sources of data and details of methods of assessment (measurement). Describe comparability of assessment methods if there is more than one group | 4,6 |
| Bias | 9 | Describe any efforts to address potential sources of bias | 6 |
| Study size | 10 | Explain how the study size was arrived at | 4 |
| Quantitative variables | 11 | Explain how quantitative variables were handled in the analyses. If applicable, describe which groupings were chosen and why | 6 |
| Statistical methods | 12 | (*a*) Describe all statistical methods, including those used to control for confounding | 6 |
|  |  | (*b*) Describe any methods used to examine subgroups and interactions | 6 |
|  |  | (*c*) Explain how missing data were addressed | NA |
|  |  | (*d*) If applicable, describe analytical methods taking account of sampling strategy | NA |
|  |  | (*e*) Describe any sensitivity analyses | NA |
| Results | | | |
| Participants | 13* | (a) Report numbers of individuals at each stage of study—eg numbers potentially eligible, examined for eligibility, confirmed eligible, included in the study, completing follow-up, and analysed | 6,7 |
|  |  | (b) Give reasons for non-participation at each stage | 7 |
|  |  | (c) Consider use of a flow diagram | NA |
| Descriptive data | 14* | (a) Give characteristics of study participants (eg demographic, clinical, social) and information on exposures and potential confounders | 7,8 |
|  |  | (b) Indicate number of participants with missing data for each variable of interest | NA |
| Outcome data | 15* | Report numbers of outcome events or summary measures | 7,8 |
| Main results | 16 | (*a*) Give unadjusted estimates and, if applicable, confounder-adjusted estimates and their precision (eg, 95% confidence interval). Make clear which confounders were adjusted for and why they were included | 7,8 |
|  |  | (*b*) Report category boundaries when continuous variables were categorized | 7,8 |
|  |  | (*c*) If relevant, consider translating estimates of relative risk into absolute risk for a meaningful time period | NA |
| Other analyses | 17 | Report other analyses done—eg analyses of subgroups and interactions, and sensitivity analyses | 7,8 |
| Discussion | | | |
| Key results | 18 | Summarise key results with reference to study objectives | 8,9 |
| Limitations | 19 | Discuss limitations of the study, taking into account sources of potential bias or imprecision. Discuss both direction and magnitude of any potential bias | 9,10 |
| Interpretation | 20 | Give a cautious overall interpretation of results considering objectives, limitations, multiplicity of analyses, results from similar studies, and other relevant evidence | 9,10 |
| Generalisability | 21 | Discuss the generalisability (external validity) of the study results | 9 |
| Other information | | | |
| Funding | 22 | Give the source of funding and the role of the funders for the present study and, if applicable, for the original study on which the present article is based | 11 |

*Give information separately for exposed and unexposed groups.

Reference: von Elm, E., Altman, D. G., Egger, M., Pocock, S. J., Gøtzsche, P. C., & Vandenbroucke, J. P. (2007). The Strengthening the Reporting of Observational Studies in Epidemiology (STROBE) statement: Guidelines for reporting observational studies. *Epidemiology*, *18*(6), 800–804. https://doi.org/10.1097/EDE.0b013e3181577654

**Supplementary table 2. Consolidated criteria for strengthening reporting of health research involving indigenous peoples: the CONSIDER statement**

| **CONSIDER Statement: Checklist Items** | |
| --- | --- |
| Governance | |
| 1. | Describe partnership agreements between the research institution and Indigenous-governing organization for the research, (e.g., Informal agreements through to MOU (Memorandum of Understanding) or MOA (Memorandum of Agreement)). |
|  | The project was a collaboration between Indigenous Māori Health and Women’s Health in the district health areas, the Breast Screen Aotearoa (BSA; national programme), Lead Providers and primary care, and supported by the National Screening Unit (at the time in the Ministry of Health).  The project was resourced under the Māori Health Pipeline, a programme of work addressing the life expectancy gap for Māori, being undertaken in two local health districts where the project took place.  Overall governance sat with a stakeholder group which included Māori Health representation (seven members of the governance group are Māori). |
| 2. | Describe accountability and review mechanisms within the partnership agreement that addresses harm minimization. |
|  | The project had the support and endorsement of Māori stakeholder organisations who had Māori staff represented on the governance group. This included the National Hauora Coalition (Indigenous Māori primary health organisation)and Te Whānau o Waipareira (urban Māori collective organisation). Non-Māori organisations also made Māori staff available for governance roles and co-design of the project and communication resources.  Formal review mechanisms in the governance group (project documentation, data agreements) were also included alongside a range of data related regulatory approvals (metropolitan Auckland data stewardship group, privacy and security groups, research office localities including Māori review).  The purpose of the project was to a undertake a datamatching process for the purposes of inviting an additional 500 unscreened or overdue Māori women to participate in the national breast screening programme, to achieve equity in breast screening DHB coverage (70%). It included:   - Providing Māori leadership and advice to facilitate the project and activities. - Ensuring Māori values and experiences informed the design and delivery in the project and activities. - Providing kaupapa Māori (Māori approach) and te ao Māori expertise and to ensure that the Māori voice and perspectives are reflected and incorporated into the design and delivery of this project. - Ensuring that the governance and way of working is aligned with Te Tiriti o Waitangi (Aotearoa New Zealand founding document between the British Crown and Māori). |
| 3. | Specify how the research partnership agreement includes protection of Indigenous intellectual property and knowledge arising from the research, including financial and intellectual benefits generated (e.g., development of traditional medicines for commercial purposes or supporting the Indigenous community to develop commercialization proposals generated from the research). |
|  | There are no commercial benefits or benefit sharing approaches related to this work. The intellectual work of the group and the Māori members and advisors is acknowledged in all project reports and authorship of any relevant publications. The project outcome contributed to informing programme and equity benefits to a national population register for breast screening, which was subsequently funded. |
| Prioritization | |
| 4. | Explain how the research aims emerged from priorities identified by either Indigenous stakeholders, governing bodies, funders, non-government organization(s), stakeholders, consumers, and empirical evidence |
|  | Māori health priorities of cancer, including breast cancer have been confirmed through national and local Māori health strategies, plans and equity analyses.  Project aims emerged out of national data which identified that despite a range of efforts over time, some BSA Lead Providers (LPs) are unable to work with all the general practices (or their organising bodies, Primary Health Organisations [PHOs]) in their area to identify Māori women to offer screening. Additionally, BSA currently does not have a population register for breast screening, and cannot systematically identify the eligible women, including those enrolled in primary care and those not enrolled. Therefore, breast screening participation relies on women being referred by their primary care provider or self-referring.  In Aotearoa New Zealand, breast screening has not achieved an equitable coverage by ethnicity, with consistently lower than the target 70% coverage for Indigenous women. While within the BSA programme Māori women have equally positive outcomes for screen detected cancers, overall there remain inequities in mortality for Māori women with late stage at diagnosis was the most important contributor to excess mortality in Māori women. |
| Relationships (Indigenous stakeholders/participants and Research team) | |
| 5. | Specify measures that adhere and honour Indigenous ethical guidelines, processes, and approvals for all relevant Indigenous stakeholders, recognizing that multiple Indigenous partners may be involved, e.g., Indigenous ethics committee approval, regional/national ethics approval processes. |
|  | Broadly the project was driven to improve outcomes for Indigenous Māori women through access to screening. This perspective included honouring Te Tiriti o Waitangi, the right to health (under the United Declaration of the Rights of Indigenous People), and the right to access high quality healthcare.  The project was approved by the BSA Programme, the Ministry of Health Data Governance Group, the local Privacy and Security Governance Group (including legal and privacy representation), the Regional Privacy Advisory Group, the Metropolitan Auckland Data Stewardship Group, the Metropolitan Auckland Clinical Governance Forum, and district locality processes at each site. All of these groups have Māori representation.  A Te Mana Raraunga Māori Data Sovereignty assessment was completed and reviewed by Māori stakeholders. Te Ara Tika, the New Zealand Health Research Council Māori ethical guidelines informed the process. |
| 6. | Report how Indigenous stakeholders were involved in the research processes (i.e., research design, funding, implementation, analysis, dissemination/recruitment). |
|  | Māori members of the governance and working group levels were involved in all aspects of the work from development of the topic, project design, methods, review of study documents, all communication collateral and were involved in review of the data analyses. |
| 7. | Describe the expertise of the research team in Indigenous health and research. |
|  | The project lead undertakes a programme of work called the Māori Health Pipeline focused on concrete interventions to address the life expectancy gap. She works in partnership with Māori academics, governance groups, advisors, Māori staff and Māori participants. She has a long standing commitment to addressing inequities and generating evidence to inform decision making. |
| Methodologies | |
| 8. | Describe the methodological approach of the research including a rationale of methods used and implication for Indigenous stakeholders, e.g., privacy and confidentiality (individual and collective) |
|  | Discussed in the governance groups and in the approval processes and outlined in the study protocol, and data management plan.  Of note, the development of the letters to women were modelled on a successful local project led by the Māori Health lead in one of the districts, and drew on Indigenous Māori values of *manaakitanga* (care for others) and *tautoko* (support). This approach was supported by the privacy committee with a small privacy commentary at the footer of the letter. |
| 9. | Describe how the research methodology incorporated consideration of the physical, social, economic and cultural environment of the participants and prospective participants. (e.g., impacts of colonization, racism, and social justice). As well as Indigenous worldviews. |
|  | With the sensitive nature of both access to identifiable data (including contact details) and the service which was breast screening, data access and management were managed in a culturally safe and appropriate manner. Activities to ensure culturally safe management included the following: Māori membership of the project working group (seven of the 11 members of the group were Māori, this included membership from an urban Māori organisation Te Whānau o Waipareira that had a Memorandum of Understanding with the local health district, membership from a Māori primary health organisation, Māori LP leadership, and Māori health commissioning leadership), Māori members of the relevant reviewing groups (who had a focus on ensuring cultural safety and equity benefit for wāhine Māori (Māori women) and on Māori data governance), review of the wording of the invitation letter including the use of Te Reo Māori (Māori language) by the district Māori health managers, a Māori equity assessment of the project via the localities process, and assessment of the project using the principals and project application tools of the Te Mana Rauranga Māori Data Sovereignty guidance. Findings were reported back to relevant Māori organisations, the Māori management network and the Ministry of Health BSA Lead Providers to systematically invite eligible women to breast screening.  Aligned with other Māori Health Pipeline projects this project used 5 contact attempts to address some of the known impacts of the social determinants of health (due to a history of colonisation, racism and structural inequities) including access barriers for Māori – the project reports on the marginal benefit of each contact attempt to improve the body of knowledge of this approach. |
| Participation | |
| 10. | Specify how individual and collective consent was sought to conduct future analysis on collected samples and data (e.g., additional secondary analyses; third-parties accessing samples (genetic, tissue, blood) for further analyses). |
|  | No samples or tissue were collected from participants. Data was collected only for the purpose of identifying women who identified as Māori ethnicity, aged between 45 and 69 years, who were enrolled with a primary care practice in one of the following four Northern health district areas. This Northern region comprises diverse ethnic populations and approximately 40% of total Aotearoa New Zealand population. The ethnicity data used were from the primary care enrolment record, routinely recorded in accordance with the Ethnicity Data Protocols for Health and Disability Sector of Aotearoa New Zealand.  The project did not require formal health ethics committee review; however, as part of the protocol preparation ethical issues were considered and documented, including mitigations, as part of good practice. These issues included privacy and confidentiality, the potential to cause distress (e.g., if contacting patients who had a recent cancer diagnosis or who had passed away), cultural safety and addressing inequities. Under the New Zealand Privacy Act (2020) and associated Health Information Privacy Code,15 the project was considered justifiable meeting an exemption (allowing waiver of consent for the project) because women who enrolled with primary care organisations sign a consent for offer of community services, including population screening. There was further support for this justification using the provisions for offer of service under Section 22F of the New Zealand Health Act, which allows health agencies to share data for the purpose of the offer of a health service. A formal Privacy Impact Assessment was undertaken. The project was approved by the BSA Programme, the Ministry of Health Data Governance Group, the local Privacy and Security Governance Group (including legal and privacy representation), the Regional Privacy Advisory Group, the Metropolitan Auckland Data Stewardship Group, the Metropolitan Auckland Clinical Governance Forum, and district locality processes at each site. The participants were informed of the project at the time of contact, with the opportunity to decline enrolment or screening, and a standardised complaints process was agreed and documented should this be required. |
| 11. | Describe how the resource demands (current and future) placed on Indigenous participants and communities involved in the research were identified and agreed upon including any resourcing for participation, knowledge, and expertise |
|  | N/A the study used data already collected |
| 12. | Specify how biological tissue and other samples including data were stored, explaining the processes of removal from traditional lands, if done, and of disposal. |
|  | N/A as above |
| Capacity | |
| 13. | Explain how the research supported the development and maintenance of Indigenous research capacity (e.g., specific funding of Indigenous researchers). |
|  | This project was a quality improvement activity rather than research. The project and project membership contributed to a potential future national process for improving access to life-saving breast screening for Māori women and for other researchers or agencies to build on further data matching opportunities to address inequities, and thereby enhance indigenous research capacity. |
| 14. | Discuss how the research team undertook professional development opportunities to develop the capacity to partner with Indigenous stakeholders? |
|  | All members have been involved in different aspects of Te Tiriti o Waitangi and cultural safety training and education. |
| Analysis and interpretation | |
| 15. | Specify how the research analysis and reporting supported critical inquiry and a strength-based approach that was inclusive of Indigenous values. |
|  | Grounding in Māori values was discussed in the research team. The Te Mana o Te Raraunga Framework Assessment Questions *Takarangi* (spiral) were used to help assess the level of sensitivity and *taonga* (presciousness/value), which supports the identification of an appropriate level of data management. The framework resembles a takarangi, consisting of two independent interwoven spirals. As you track along either the *tapu* (sacred) or *noa* (common) spiral you pass through each of the four planes representing core Māori concepts relevant to the management of data. These concepts inform the questions that relate to an assessment of the data, an assessment of the data use, and an assessment of the data users. |
| Dissemination | |
| 16. | Describe the dissemination of the research findings to relevant Indigenous governing bodies and peoples. |
|  | The study results were disseminated widely to all relevant stakeholders including Māori stakeholders and governance groups. |
| 17. | Discuss the process for knowledge translation and implementation to support Indigenous advancement (e.g., research capacity, policy, investment). |
|  | This study was reported to the BSA programme and National Screening Unit to inform a potential national approach to datamatching in order to identify unenrolled and overdue women in the BSA and other screening programmes. |

Reference: Huria, T., Palmer, S. C., Pitama, S., Beckert, L., Lacey, C., Ewen, S., & Smith, L. T. (2019). Consolidated criteria for strengthening reporting of health research involving indigenous peoples: The CONSIDER statement. *BMC Medical Research Methodology*, *19*(1). https://doi.org/10.1186/s12874-019-0815-8

**Supplementary table 3. Invitation letter**

Date NHI Number:

Name

Address Line1

Address Line 2

Address Line 3

**Kia tere horo te haumaru mōu***[Seize the opportunity to keep yourself safe]*

Tēnā koe e XXXX,
*[Greetings]*

As a patient of **(xxx GP/Clinic),** you are invited to participate in BreastScreen Aotearoa’s ***free*** national breast screening programme for women aged between 45 and 69. Your doctor will be informed of your test results (if you choose).

We recommend that you have regular breast screening mammograms, as early detection of Breast Cancer saves lives.

BreastScreen Aotearoa has screening centres across Auckland and mobile screening units that move around the region.

Support to attend appointments can be arranged if you need transport.

Appointments are available outside working hours and on weekends if this is easier for you to attend.

**Free phone** **0800 270 200 to make an appointment**

If you have any questions, please call the free phone number above.

Yours sincerely

Xxxxx

Manager, BreastScreen (xxx,xxx)

This initiative uses secure access to information held by BreastScreen Aotearoa, Primary Health Organisations and the Ministry of Health to identify and contact women not currently benefitting from the free national breast screening programme.

-- end of letter --

Note: The letter content was based on a combination of invitation letters in primary care or recall letters from the programme, a Māori led screening engagement for cervical screening in one of the LP areas, and legal advice regarding privacy statements. Women could opt out through notifying the LP via phone number – it would be noted on their file. When women called, they were given further information about BSA and when they enrolled (on enrolment form and process) and were screened, they were provided with the standard BSA information about the programme, screening, benefits and harms etc.
